# Supplementary material for: Comparative genomics provides new insights into the diversity, physiology, and sexuality of the only industrially exploited tremellomycete: Phaffia rhodozyma
Source: BMC Genomics. 2016 Nov 9;17:901. doi: 10.1186/s12864-016-3244-7 (PMC5103461; doi:10.1186/s12864-016-3244-7)
Supplement: Additional file 6: — List of orphan genes with links to PFAM (related to Additional file 1: Table S1). (ZIP 1428 kb) [file 12864_2016_3244_MOESM6_ESM.zip › BLAST_HTML_FTR/G02893_P.html]

BLAST Search Results


```
BLASTP 2.2.27+


Reference:
Stephen F. Altschul, Thomas L. Madden, Alejandro A. Schäffer,
Jinghui Zhang, Zheng Zhang, Webb Miller, and David J. Lipman (1997),
"Gapped BLAST and PSI-BLAST: a new generation of protein database
search programs", Nucleic Acids Res. 25:3389-3402.


Reference for
composition-based statistics:
Alejandro A. Schäffer, L. Aravind, Thomas L. Madden, Sergei
Shavirin, John L. Spouge, Yuri I. Wolf, Eugene V. Koonin, and
Stephen F. Altschul (2001), "Improving the accuracy of PSI-BLAST
protein database searches with composition-based statistics and
other refinements", Nucleic Acids Res. 29:2994-3005.


Database: nr
           71,551,133 sequences; 26,053,659,533 total letters


Query= G02893_P

Length=126
                                                                      Score     E
Sequences producing significant alignments:                          (Bits)  Value

emb|CED83490.1|  hypothetical protein [Xanthophyllomyces dendrorh...   236    4e-77
ref|XP_013137175.1|  PREDICTED: mitotic spindle assembly checkpoi...  42.0    0.060
emb|CED82385.1|  hypothetical protein [Xanthophyllomyces dendrorh...  35.8    6.5  


 >emb|CED83490.1| hypothetical protein [Xanthophyllomyces dendrorhous]
Length=125

 Score =  236 bits (601),  Expect = 4e-77, Method: Compositional matrix adjust.
 Identities = 125/125 (100%), Positives = 125/125 (100%), Gaps = 0/125 (0%)

Query  1    MPLITRKNTPPSFVIGQSASFTQPTQQMSSPPVIILTPPPSDMPTPPADTQLSPTGPSLV  60
            MPLITRKNTPPSFVIGQSASFTQPTQQMSSPPVIILTPPPSDMPTPPADTQLSPTGPSLV
Sbjct  1    MPLITRKNTPPSFVIGQSASFTQPTQQMSSPPVIILTPPPSDMPTPPADTQLSPTGPSLV  60

Query  61   RRVSGASSSASSKSYDVSYFLPSRRSRRPSSPPGPIPVRLRNSKLILDQNSPFRARALGL  120
            RRVSGASSSASSKSYDVSYFLPSRRSRRPSSPPGPIPVRLRNSKLILDQNSPFRARALGL
Sbjct  61   RRVSGASSSASSKSYDVSYFLPSRRSRRPSSPPGPIPVRLRNSKLILDQNSPFRARALGL  120

Query  121  DLVVP  125
            DLVVP
Sbjct  121  DLVVP  125


>ref|XP_013137175.1| PREDICTED: mitotic spindle assembly checkpoint protein MAD1 [Papilio 
polytes]
Length=721

 Score = 42.0 bits (97),  Expect = 0.060, Method: Composition-based stats.
 Identities = 27/91 (30%), Positives = 41/91 (45%), Gaps = 13/91 (14%)

Query  34   IILTPPPSDMPTPPADTQLSPTGPSLVRRVSGASSSASSKSYDVSYFL-PSRRSRRPSSP  92
            +I T PP D  +  +   LS +  S+   +S   S+    S D S  L P +R RR SS 
Sbjct  20   VINTDPPKDKLSASSKLNLSNSDASIKESISNLLSTKRKSSLDTSDLLTPEKRPRRESST  79

Query  93   PGPIPVRLRNSKLILDQNSPFRARALGLDLV  123
              P+P             SP+ A+ + +DL+
Sbjct  80   NSPVP------------PSPWEAKKMKIDLI  98


>emb|CED82385.1| hypothetical protein [Xanthophyllomyces dendrorhous]
Length=275

 Score = 35.8 bits (81),  Expect = 6.5, Method: Compositional matrix adjust.
 Identities = 16/27 (59%), Positives = 20/27 (74%), Gaps = 0/27 (0%)

Query  92   PPGPIPVRLRNSKLILDQNSPFRARAL  118
            PPGPIPVRL+NS  +   +SPF  RA+
Sbjct  86   PPGPIPVRLQNSLYLQSSSSPFYQRAM  112


Lambda      K        H        a         alpha
   0.318    0.133    0.395    0.792     4.96 

Gapped
Lambda      K        H        a         alpha    sigma
   0.267   0.0410    0.140     1.90     42.6     43.6 

Effective search space used: 640180337412


  Database: nr
    Posted date:  Sep 23, 2015 12:05 AM
  Number of letters in database: 26,053,659,533
  Number of sequences in database:  71,551,133


Matrix: BLOSUM62
Gap Penalties: Existence: 11, Extension: 1
Neighboring words threshold: 11
Window for multiple hits: 40
```
